# The “Abdominal Circulatory Pump”: An Auxiliary Heart during Exercise?

**DOI:** 10.3389/fphys.2015.00411

**Published:** 2016-01-07

**Authors:** Barbara Uva, Andrea Aliverti, Dario Bovio, Bengt Kayser

**Affiliations:** ^1^Institute of Sport Sciences and Department of Physiology, Faculté de Biologie et de Médecine, Université de LausanneLausanne, Switzerland; ^2^Dipartimento di Elettronica, Informazione e Bioingegneria, Politecnico di MilanoMilano, Italy

**Keywords:** exercise, cardiac output, venous return, splanchnic circulation, inferior vena cava

## Abstract

Apart from its role as a flow generator for ventilation the diaphragm has a circulatory role. The cyclical abdominal pressure variations from its contractions cause swings in venous return from the splanchnic venous circulation. During exercise the action of the abdominal muscles may enhance this circulatory function of the diaphragm. Eleven healthy subjects (25 ± 7 year, 70 ± 11 kg, 1.78 ± 0.1 m, 3 F) performed plantar flexion exercise at ~4 METs. Changes in body volume (ΔV_b_) and trunk volume (ΔV_tr_) were measured simultaneously by double body plethysmography. Volume of blood shifts between trunk and extremities (V_bs_) was determined non-invasively as ΔV_tr_-ΔV_b_. Three types of breathing were studied: spontaneous (SE), rib cage (RCE, voluntary emphasized inspiratory rib cage breathing), and abdominal (ABE, voluntary active abdominal expiration breathing). During SE and RCE blood was displaced from the extremities into the trunk (on average 0.16 ± 0.33 L and 0.48 ± 0.55 L, *p* < 0.05 SE vs. RCE), while during ABE it was displaced from the trunk to the extremities (0.22 ± 0.20 L *p* < 0.001, *p* < 0.05 RCE and SE vs. ABE respectively). At baseline, V_bs_ swings (maximum to minimum amplitude) were bimodal and averaged 0.13 ± 0.08 L. During exercise, V_bs_ swings consistently increased (0.42 ± 0.34 L, 0.40 ± 0.26 L, 0.46 ± 0.21 L, for SE, RCE and ABE respectively, all *p* < 0.01 vs. baseline). It follows that during leg exercise significant bi-directional blood shifting occurs between the trunk and the extremities. The dynamics and partitioning of these blood shifts strongly depend on the relative predominance of the action of the diaphragm, the rib cage and the abdominal muscles. Depending on the partitioning between respiratory muscles for the act of breathing, the distribution of blood between trunk and extremities can vary by up to 1 L. We conclude that during exercise the abdominal muscles and the diaphragm might play a role of an “auxiliary heart.”

## Introduction

The cyclical swings in intrathoracic and abdominal pressures caused by breathing influence cardiovascular function at rest and during exercise. The effects on venous return and blood redistribution from and to the limbs have been described qualitatively, both in humans and in animal models (Harms et al., 1997; Aliverti and Macklem, 2001; Aliverti et al., 2004, 2010; Miller et al., 2005a). The splanchnic region contains a large venous reservoir capable of rapidly delivering blood toward the right heart upon increases in transdiaphragmatic pressure, such as during exercise or expulsive maneuvers (Flamm et al., 1990; Aliverti et al., 2009). Flamm et al. (1990) estimated a reduction of splanchnic blood volume by 20% from rest to exercise and a corresponding increase in rib cage blood volume.

Aliverti et al. quantified the dynamics of blood shifting between the trunk and the extremities during quiet breathing and voluntary expulsive maneuvers, consisting of simultaneous contraction of the diaphragm and the abdominal muscles (Aliverti et al., 2009, 2010). The diaphragm, acting both on abdominal and pleural pressures, clearly had a circulatory function in addition to its ventilatory one. Through its effects on abdominal pressure it produced breath-by-breath oscillation of inferior vena cava blood flow, confirming the cyclical effect of intrathoracic and abdominal pressure swings on venous return, as shown before (Miller et al., 2005b).

The variations in abdominal pressure during quiet diaphragmatic breathing at rest, result in a blood volume of ~50–75 ml to be shifted in and out of the splanchnic reservoir during each breath (Aliverti et al., 2009). With expulsive maneuvers, which increased abdominal pressure up to 140 cmH_2_O, more than 600 ml of blood was shifted out of the splanchnic venous compartment while femoral venous return was halted (Aliverti et al., 2009). The splanchnic region may thus play the role of an auxiliary heart, and it was hypothesized that such a role might be of even greater importance during physical exercise, when metabolic demand leads to an increase in cardiac output and larger abdominal and transdiaphragmatic pressure swings impact on venous return (Aliverti et al., 2010).

We therefore studied 11 healthy subjects during exercise, while breathing in 3 different ways: (a) spontaneously (hereafter referred to as “spontaneous mode”); (b) predominantly using the inspiratory rib cage muscles (“rib cage mode”); and (c) predominantly using the abdominal muscles during expiration and the diaphragm during inspiration (“abdominal mode”). We looked at the impact of these breathing modes during moderate exercise, on blood shifts between the trunk and the extremities. The volume of blood shifting between the trunk and the extremities was continuously measured using double body plethysmography (DBP). Changes in body volume (ΔV_b_) and trunk volume (ΔV_tr_) were simultaneously tracked using whole body plethysmography (WBP) and opto-electronic plethysmography (OEP). This allowed quantification of blood shifting between trunk and extremities, calculated as the difference between these two simultaneous measurements (V_bs_ = ΔV_tr_ − ΔV_b_). We hypothesized that during exercise the three breathing modes would differentially impact on intrathoracic and abdominal pressure variations, and therefore cardiovascular function. In particular, breathing with the diaphragm and the abdominal muscles would primarily act on abdominal pressure, while using the rib cage muscles would primarily act on pleural pressure. We expected that during exercise, compared to resting, the volume of blood displaced in and out the trunk would increase significantly and that the direction and amount of this blood displacement would depend on the relative predominance of the action of diaphragm, rib cage and abdominal muscles during the three breathing modes.

## Methods

### Subjects

Eleven healthy subjects (25 ± 7 year, mean ± SD, 70 ± 11 kg, 1.78 ± 0.10 m, 3 women), recruited among laboratory personnel, volunteered for the experiments. Most were experienced in doing complex respiratory maneuvers; all of them were instructed in detail at the beginning of each maneuver. The subjects' characteristics are shown in Table 1. The research ethics committee of the INRCA Hospital approved the research protocol and informed written consent was obtained from each participant in accordance with the Declaration of Helsinki.

**Table 1:** **Subjects' characteristics**.

**Subj No.**	**Age (years)**	**Sex**	**Weight (kg)**	**Height (cm)**	**BMI (kg/m^2^)**	**BSA (m^2^)**
1	23	M	58	168	20.55	1.66
2	21	M	62	194	16.47	1.89
3	23	M	77	178	24.30	1.95
4	22	F	62	180	19.14	1.79
5	22	M	70	173	23.39	1.83
6	45	M	75	178	23.67	1.93
7	24	M	83	183	24.78	2.05
8	22	M	83	181	25.34	2.04
9	24	F	50	167	17.93	1.55
10	27	F	58	163	21.83	1.62
11	28	M	80	194	21.26	2.11

### Volume measurements

To quantify blood volume shifting between the trunk and the extremities, we used DBP using a WBP with walls made of transparent cast acrylic sheet (20 mm) (Aliverti et al., 2009). WBP allowed monitoring body volume changes (ΔV_b_), resulting from the gas flowing in and out of the lungs, and the simultaneous changes in compression and decompression of thoracic gas (Goldman et al., 2005). OEP allowed quantifying the changes of trunk volume (ΔV_tr_), resulting from the lung volume changes plus any blood shifting between the trunk and the extremities (V_bs_). By subtracting the two independent measurements of ΔV_tr_ and ΔV_b_ we thus obtained V_bs_. ΔV_b_ was measured continuously with the subjects sitting comfortably in the WBP. The flow in and out of the WBP due to changes in body volume was measured by a pneumotachometer mounted on the top of the box, connecting the box's interior with the exterior. From this flow signal ΔV_b_ was obtained by mathematical integration.

ΔV_tr_ was simultaneously measured by OEP, using 89 retro-reflective markers placed on the front and back of the chest wall of the subject (Aliverti et al., 2004). Eight cameras captured the markers' positions at a frequency of 60 Hz and the three-dimensional coordinates of the markers were calculated by stereo-photogrammetry using a 3D calibrated motion analyzer. The chest was modeled in two compartments: the rib cage and the abdomen. The boundary between the rib cage and abdomen was fixed along the lower costal margin, as described in detail in the previous studies (Aliverti et al., 2001, 2003, 2004). Total chest wall volume (V_cw_) was calculated as the sum of rib cage and abdominal volume (V_rc_ and V_ab_ respectively). Flow at the mouth was measured using a hot wire anemometer, which measured gas velocity in a tube of known cross-sectional area (Sensormedics Vmax, Yorba Linda, USA), connecting the subject to the exterior of the box. Both flow meters were calibrated with a 3 L syringe.

Temperature and humidity inside the box were continuously controlled with a multi-sensor device (SHT11, Sensirion AG, Zuerich, Switzerland) based on a hygroscopic polymer as a humidity sensitive element and a semiconductor as a temperature-sensitive element. Eight Peltier cells (ETH-127-14-15-RS, Global Component Sourcing, Central, Hong Kong, China) were interfaced with a set of finned heat sinks (Hyper Series, Cooler Master Corporation, Zhonghe District, New Taipei City, Taiwan) and placed inside and outside the box. The air conditioning system was placed at the bottom of the body box out of the line of sight between the OEP cameras and the trunk, to allow the correct measurement of V_tr_, and also to avoid influencing the pneumotachograph placed on the top of the box. Pilot testing in preparation for the experiments showed that this conditioning system allowed keeping temperature quite constant inside the WBP even during exercise, but not relative humidity, which led to integration drift.

### Correction for integration drift

In clinical practice WBP is routinely used for pulmonary function diagnostic tests that last so short that temperature and humidity changes can be neglected. Conversely, if prolonged volume measurements are needed, for example during an exercise test, increases in temperature and humidity, caused by the subject, cause substantial thermal drift (Goldman et al., 2005). In spite of our air conditioning system, which kept air temperature in the body-box almost constant, a consistent non-linear drift of volume occurred during exercise. We used discrete wavelet transformation (DWT) (von Borries et al., 2005) to remove this drift from the signals, because DWT provides a multi-resolution signal decomposition allowing the analysis of the signal at different frequency bands with different resolutions (Rioul and Vetterli, 1991; Samar et al., 1995). The algorithm used is based on a discrete wavelet transformation using high-pass and low-pass filters (scaling function) applied iteratively to the signal, which is progressively down sampled. The low-pass filter gives the new scaling coefficients while the high-pass filter gives the wavelet coefficients in two orthogonal subspaces. The scaling and wavelet coefficient correspond to the low and high frequency components of the input. The decomposition is then repeated with the analysis filter over the scaling coefficient a number of times depending on the bandwidth of the baseline drift (Rioul and Vetterli, 1991; Samar et al., 1995; von Borries et al., 2005). We used a second order discrete biohortogonal function as mother wavelet, because it best fit our signal, and because it was linear in phase, symmetrical, and had good local properties. After decomposition the input signal was then reconstructed using the same family of wavelets but forcing to zero the low frequency coefficient, representing the thermal drift. This allowed removing the thermal drift without introducing distortion and protecting the shape of the original signal. In order to choose the correct level of wavelet decomposition and to verify if the resulting volume signal was well filtered, we did a linear regression between the first phase of quiet breathing of the filtered signal and the same part of quiet breathing of the chest wall volume, acquired by OEP, which was by definition not affected by thermal drift. During spontaneous quiet breathing these two signals should be aligned and in phase (Aliverti et al., 2009, 2010). For each trial the level with the highest R^2^ resulting from this procedure was then used.

### Exercise testing

The subjects were studied while sitting inside the WBP during pre-exercise baseline and submaximal constant workload exercise. We used a custom-made stepper, the only possible exercise solution because of the dimensions of the WBP. It consisted of an electric motor used as a generator, connected to two pedals via a chain-sprocket system to transfer the movement of the pedals to the axis of the motor. The current generated was used to calculate the instantaneous power developed by the subject measuring the voltage drop over a known resistance using the formula P = V^2^/R. Subjects were asked to step at a frequency of about 30 cycles/min, which provided an average workload of about 64 W.

Oxygen uptake (V′O_2_), carbon dioxide output (V′CO_2_) and end-tidal CO_2_ pressure (P_ET_CO_2_) were continuously measured on a breath-by-breath basis using a respiratory gas analyzer (Sensormedics Vmax, Yorba Linda, USA). O_2_ and CO_2_ concentrations at the mouth were measured by paramagnetic and infrared gas analyzers, integral part of the system, calibrated with gases of known composition. The flow meter was calibrated with a 3 L syringe. For each subject, data are presented as the mean of at least 10 breaths collected pre-exercise and the last part of the three exercise bouts.

### Protocol

**Figure 1:**
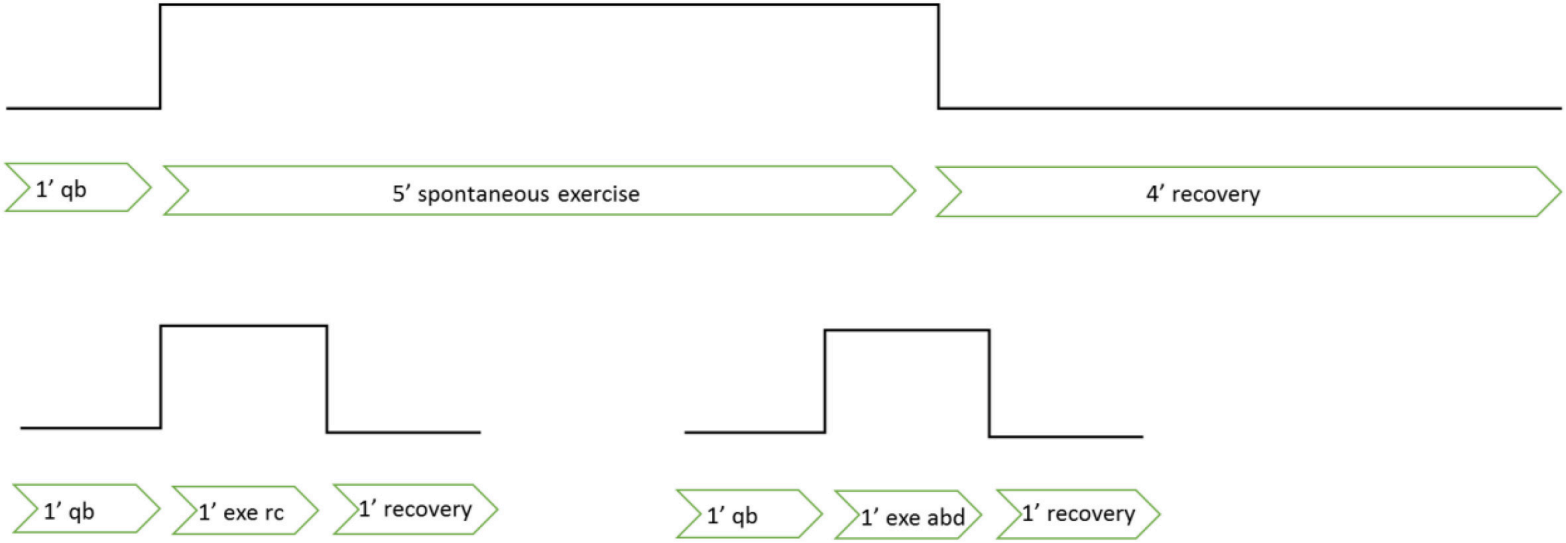
**Description of the experimental protocol**. qb, quite breathing; ex, exercise; rc, rib-cage breathing mode; abd, abdominal breathing mode.

The subjects were asked to breathe during the exercise tests using the three prescribed different modes: “spontaneous,” “rib cage,” and “abdominal,” performed in separate trials. In spontaneous mode, after 1 min of sitting baseline (quiet breathing during rest, while sitting in the WBP), they engaged in 5 min of stepping exercise while breathing spontaneously, followed by 4 min of recovery (no exercise) (see Figure 1). After instructions and some practice runs they then performed several different trials for each breathing mode. Rib cage breathing mode consisted of voluntary emphasizing inspiratory rib cage breathing, which, when effectuated correctly, resulted in ribcage volume changes over a breathing cycle while keeping abdominal volume constant. Abdominal breathing mode consisted of voluntary abdominal expiration, resulting in a decrease of abdominal volume accompanied by a simultaneous increase of abdominal pressure, followed by diaphragm contraction for inspiration, while keeping the rib cage configuration invariant. Since abdominal and rib cage breathing modes were difficult to sustain, we shortened these to 1 min, long enough for the larger initial cardiovascular and metabolic changes to occur, while short enough to allow the subjects to correctly perform the exercise. Thus, after 1 min of quiet breathing at rest, rib cage or abdominal mode exercise were executed for 1 min, followed by 1 min of recovery. Subjects did each exercise trial a minimum of one time, recovering between trials. The WBP was opened in between trials to reestablish the initial thermodynamic conditions. The order of the three breathing modes was randomized within and between subjects.

### Data analysis

**Figure 2:**
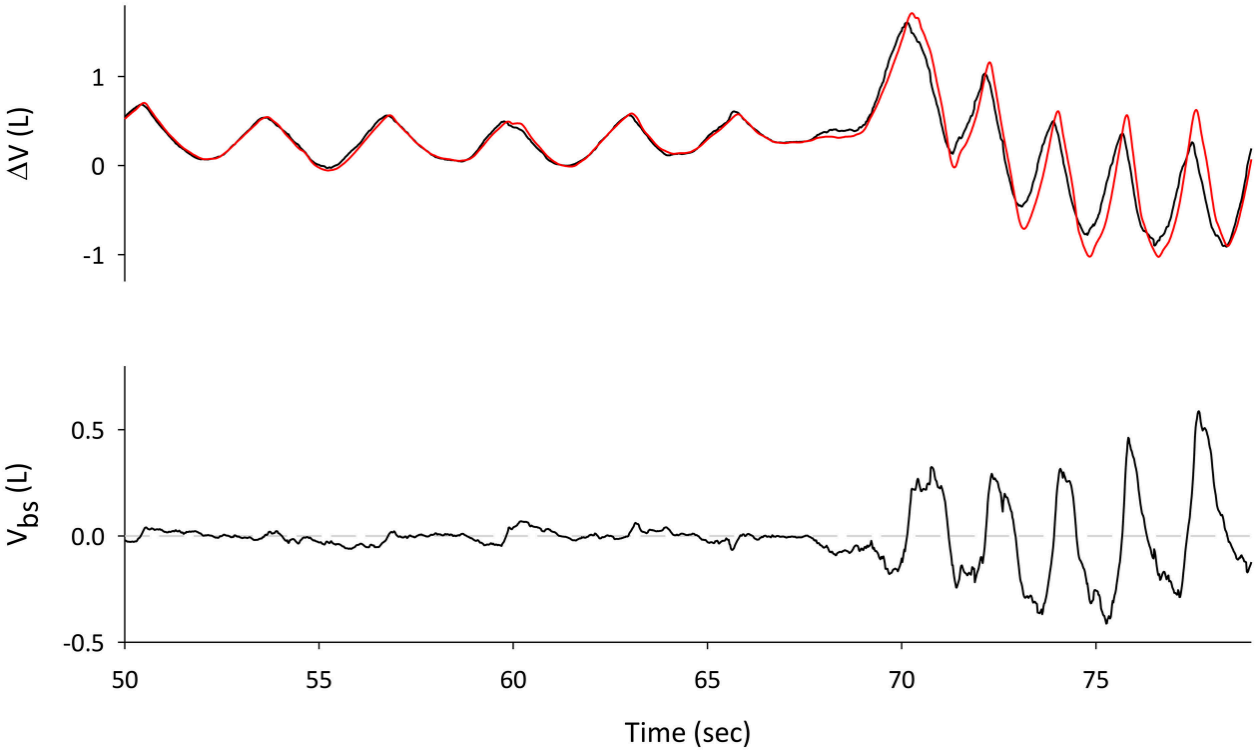
**Traces during spontaneous pre-exercise breathing**. **Top Panel**: changes in Lungs volume, measured with WBP (V_L_, red line) and Trunk volume derived from OEP (V_tr_, black line). The difference between V_tr_ and V_L_ gives the volume of the blood shifted between the splanchnic vasculature and the extremities (**bottom panel**).

**Figure 3:**
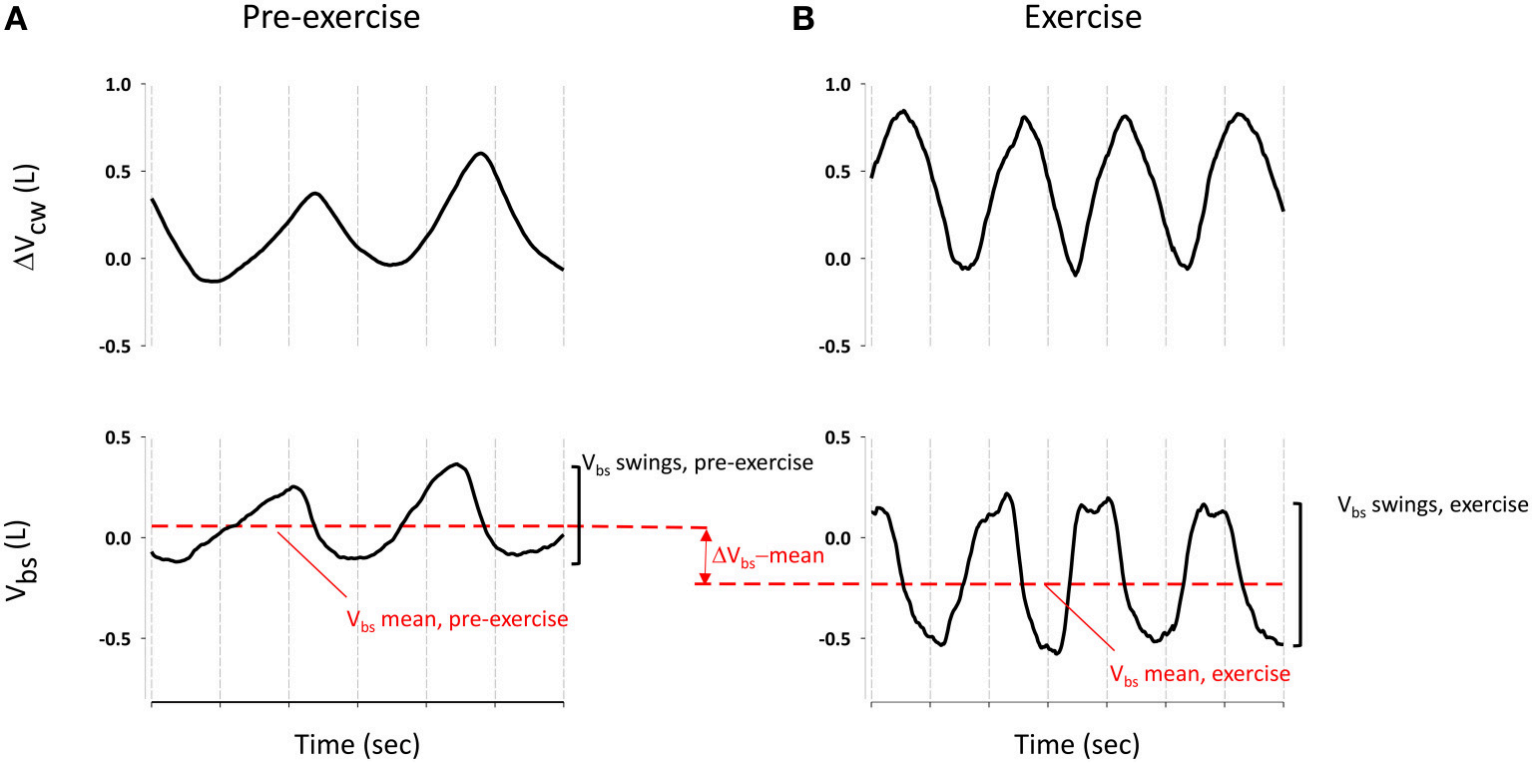
**Blood shift volume and chest wall volume variation during pre-exercise breathing (A) and an example of spontaneous breathing mode during exercise (B)**. ΔV_bs_-mean was calculated as difference between V_bs_ mean (red dashed line) during exercise and pre-exercise. V_bs_ swings were calculated as difference between the maximum and the minim values during each breathing of quiet breathing and exercise respectively.

Pre-exercise and exercise phases were analyzed in terms of volume changes and blood shifts. The analysis of V_bs_, obtained by subtracting the ΔV_tr_ and ΔV_b_ (see Figure 2), focused on two different parameters: V_bs_ swings, defined as the difference of maximum and minimum values within each breath, averaged over at least 10 breaths, pre-exercise and during the three exercise conditions, and ΔV_bs_-mean, i.e., the difference between average V_bs_ pre-exercise and during exercise (see Figure 3). The effects of different exercise breathing patterns on blood shift and volume changes were assessed by a generalized linear mixed-model (repeated measurements analysis of variance) since the missing values for some of the subjects participating to the study precluded the use of simple ANOVA. When the outcome data were not normally distributed, we used the inverse Gaussian distribution, the closest available theoretical distribution to fit the sample. The breathing modes (spontaneous, rib cage, and abdominal), the phase of exercise (quiet breathing vs. exercise) and their interactions were defined as fixed effects in the model. The subjects' variability was used as random effect. Pairwise comparisons were carried out with Holm Sidak's *post-hoc* test. The significance level was set at *P* < 0.05. All statistical analyses were carried out using SPSS, version 21 (IBM Corp., Armonk, NY, USA). Data are expressed as mean ± SD unless otherwise specified.

## Results

### Exercise

The square-wave submaximal stepping exercise was performed at about 30 cycles/min and an average power output of 64 W. Individual metabolic equivalent of task (MET) values were calculated by dividing the V′O_2_, reached during exercise between the third and fourth minute while breathing spontaneously, by 3.5 ml kg^−1^ min^−1^. The exercise intensity corresponded to an average of 4.3 METs, indicating low to moderate intensity exercise (3–6 METs). Even though the stepping effort was equivalent in the three modes, because of the shorter durations of rib cage and abdominal breathing modes compared to spontaneous mode, steady state gas exchange was not reached in rib cage and abdominal modes (duration of exercise 1 min).

Ventilatory parameters for pre-exercise and exercise are shown in Table 2. Exercise significantly increased V′O_2_ and V′CO_2_ compared to pre-exercise condition. V′O_2_ increased from pre-exercise by 11.1 ± 3.9, 8.5 ± 2.4, and 6.8 ± 2.6 ml kg^−1^ min^−1^ (all *p* < 0.001), for spontaneous, rib cage and abdominal mode, respectively. V′CO_2_ increased during exercise (*p* < 0.001) mostly during spontaneous breathing mode; 12.8 ± 3.6, 7.5 ± 1.4, 6.0 ± 1.9 ml kg^−1^ min^−1^ for spontaneous, rib cage and abdominal mode, respectively (*p* < 0.001, *p* < 0.01, spontaneous vs. abdominal and rib cage exercise, respectively). P_ET_CO_2_ during spontaneous breathing was higher compared to pre-exercise, rib cage mode and abdominal mode (33.7 ± 3.8, 40 ± 4.3, 31.1 ± 6.0, 33.3 ± 4.6 mmHg, for pre-exercise, spontaneous, rib cage and abdominal modes, respectively. *p* < 0.001 for pre-exercise and rib cage, *p* < 0.01 for abdominal). The baseline ventilation, P_ET_CO_2_ and RQ indicated some hyperventilation related to the experimental conditions.

**Table 2:** **Ventilatory parameters at pre-exercise baseline and during exercise, calculated at the same relative intensity for 5′ spontaneous breathing, rib cage breathing, and abdominal breathing respectively**.

	**Pre-exercise**	**Exercise**		
		**5′ spontaneous breathing**	**1′ rib cage breathing**	**1′ abdominal breathing**
V′O_2_/kg (ml/Kg/min)	4.0±1.5	15.2±3.9^*^	12.2±2.5^*^	11.6±2.0^*^
V′CO_2_/kg (ml/Kg/min)	4.3±1.8	17.4±4.1^*^	11.1±2.5^*^^#^	11.2±2.0^*^^#^
RQ	1.0±0.2	0.9±0.1	1.2±0.2^#^	1.1±0.2
VE (L/min)	14.4±4.3	31.5±7.8^*^	42.2±15.1^*^	37.7±13^*^
PETCO_2_ (mmHg)	33.7±3.8	40±4.3^*^	31.1±6.0^#^	33.3±4.6^#^

Values are presented as mean ± SD;

* p < 0.05 vs. pre-exercise;

# *p < 0.05 vs. 5′ spontaneous breathing mode. V′O_2_ and V′CO_2_ individual values are given in the text*.

Exercise significantly increased ventilation, which passed from 14.4 ± 4.3 L/min at pre-exercise baseline to 31.5 ± 7.8, 42.2 ± 15.1, and 37.7 ± 13 L/min (all *p* < 0.001), during spontaneous, rib cage, and abdominal mode, respectively.

**Figure 4:**
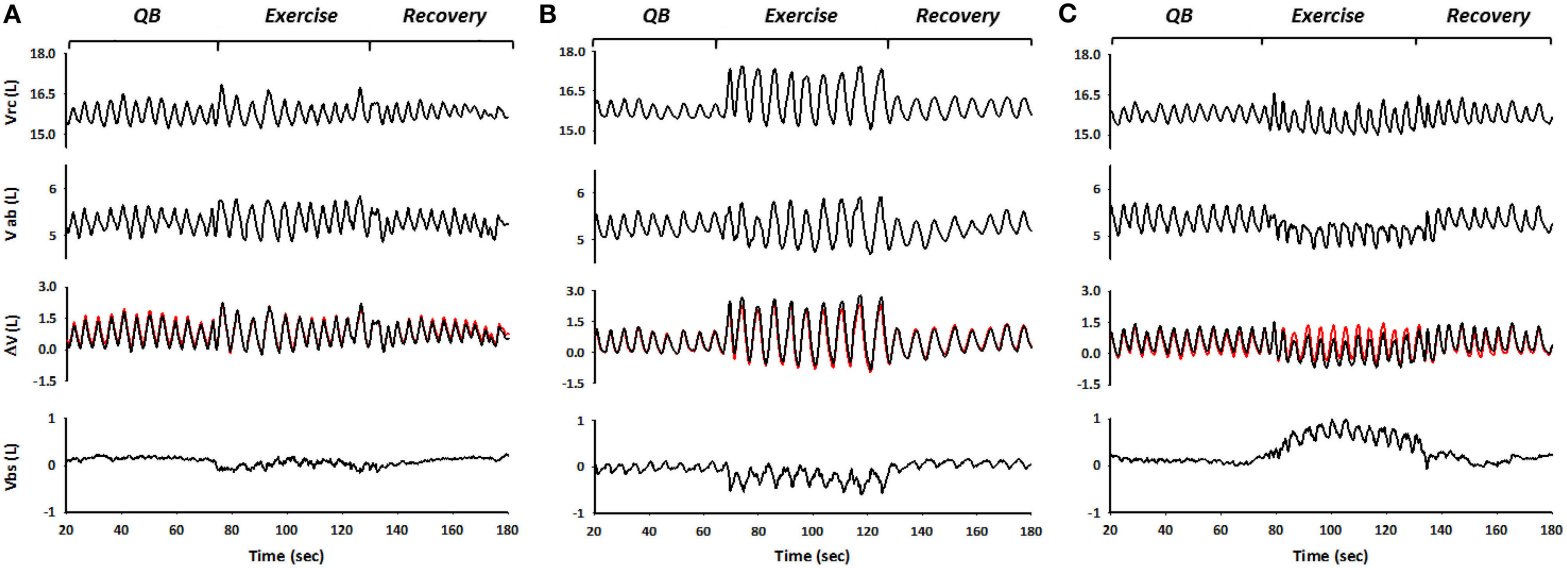
**The figure shows the three different breathing modes during exercise performed by the subjects, spontaneous (A), rib cage (B), and abdominal mode (C) respectively**. From top to bottom: OEP measured Rib cage volume (V_rc_), abdominal volume (V_ab_), derived from OEP, WBP measured changes in body volume variation and thus in lung volume variations (V_b_), superimposed in red on V_cw_ variations and blood shifts between trunk and extremities (V_bs_) calculated as the differences between ΔV_cw_ and ΔV_b_.

Representative traces of spontaneous, rib cage, and abdominal breathing modes during exercise are shown in Figure 4. From top to bottom are depicted rib cage volume (V_rc_), abdominal volume (V_ab_), and trunk volume (V_tr_), all three obtained with OEP (black lines). Superimposed in red on V_tr_ is shown WBP-measured body volume (V_b_), the difference with V_tr_ representing blood shifting (V_bs_), shown in the bottom panels.

When the subject inspired using predominantly her/his rib cage muscles—i.e., during rib cage breathing mode—end-inspiratory rib cage volume increased, while abdominal volume remained approximately constant. This resulted in blood being displaced from the extremities to the trunk, presumably to the rib cage, as illustrated by negative values of V_bs_ (Figure 4B). Conversely, during exercise while in abdominal breathing mode, end-expiratory abdominal volume decreased while the blood shifted from the trunk to the extremities, as indicated by the positive values of V_bs_ (Figure 4C).

### Chest wall volumes

**Figure 5:**
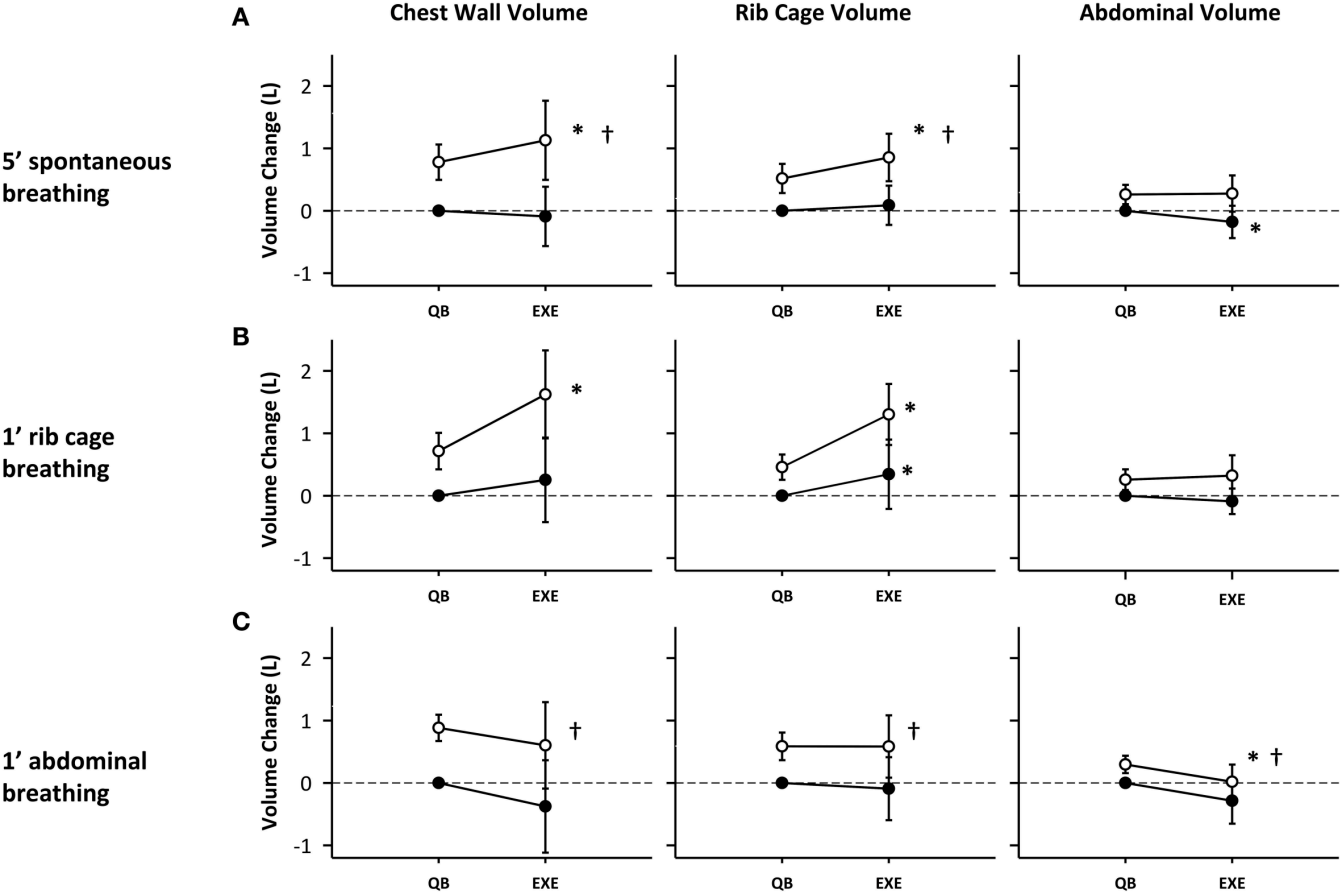
**Compartmental and total chest wall volume changes during different exercise breathing modes: (A) 5′ spontaneous breathing; (B) 1′ rib cage breathing; (C) 1′ abdominal breathing mode**. White circles: end-inspiration volume; Black circles: end-expiration volume; Mean values and standard deviation bars are shown. ^*^p < 0.05 vs. pre-exercise; ^†^*p* < 0.05 vs. rib cage breathing mode.

Figure 5 depicts the rib cage, abdominal and total chest wall end-expiratory volumes (black circles) and end-inspiratory volumes (white circles), at baseline and during exercise for each of the breathing modes. Exercise induced an increase in chest wall tidal volume (V_T_), defined as the difference of end-inspiratory volume and end-expiratory volume, for all breathing modes (0.66 ± 0.45, 0.76 ± 0.73, and 0.65 ± 0.63 L, *p* < 0.001, *p* < 0.01, *p* < 0.01, for spontaneous rib cage and abdominal mode respectively) as shown in Figure 5. In spontaneous mode, the increase in V_T_ was reached by an increase of end-inspiratory rib cage volume (V_rcEI_) and a concomitant decrease in end-expiratory abdominal volume (V_abEE_). V_rcEI_ increased on average by 0.37 ± 0.28 L (*p* < 0.01) while V_abEE_ decreased on average by 0.21 ± 0.20 L (*p* < 0.05) compared with pre-exercise baseline.

In rib cage breathing mode chest wall tidal volume significantly increased (*p* < 0.01), mostly due to an increase in end-inspiratory rib cage volume (0.88 ± 0.39 L, *p* < 0.001), while end-expiratory abdominal volume was kept nearly constant (0.09 ± 0.2 L, p = n.s.). No significant differences were found in V_cwEE_ and V_cwEI_ during abdominal exercise. The increase in tidal volume was due to a decrease in V_abEE_ (0.41 ± 0.16 L, *p* < 0.05), while V_rcEI_ was kept approximately constant (0.15 ± 0.27 L, p = n.s.).

Rib cage breathing mode significantly increased V_rcEI_ (0.84 ± 0.39 L) during exercise compared to spontaneous and abdominal breathing modes (0.41 ± 0.26, 0.16 ± 0.27 L, *p* < 0.05 and *p* < 0.01, respectively).

### Blood shifts

**Figure 6:**
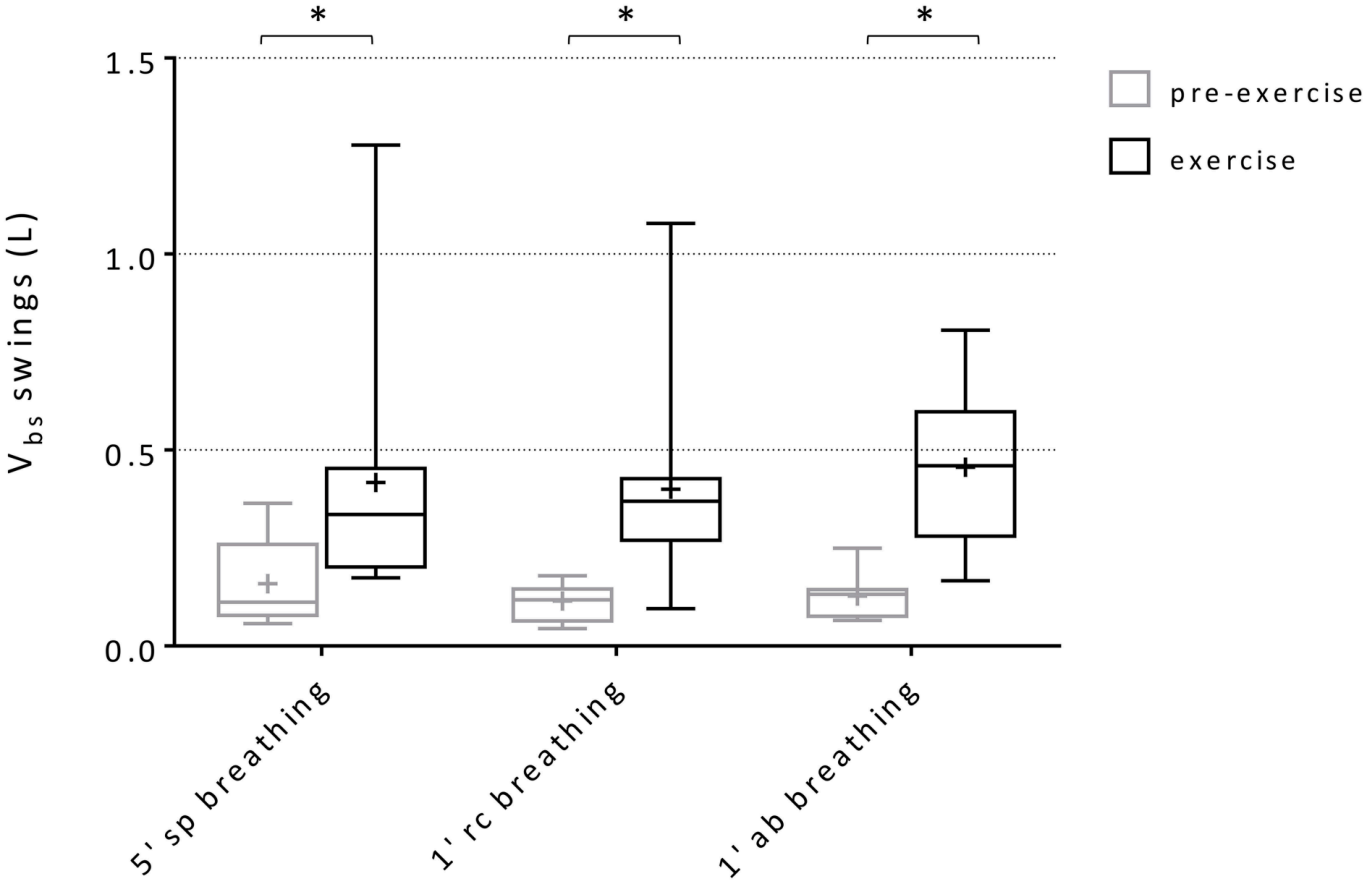
**Box-and-whisker plot displaying V_bs_ swings during the three breathing modes**. Black boxes: pre-exercise; Gray boxes: exercise. Central line, box and whisker limits represent median, interquartile range and minimum and maximum values of the data, respectively. ^+^ represents the mean value ^*^p < 0.01 vs. pre-exercise.

**Figure 7:**
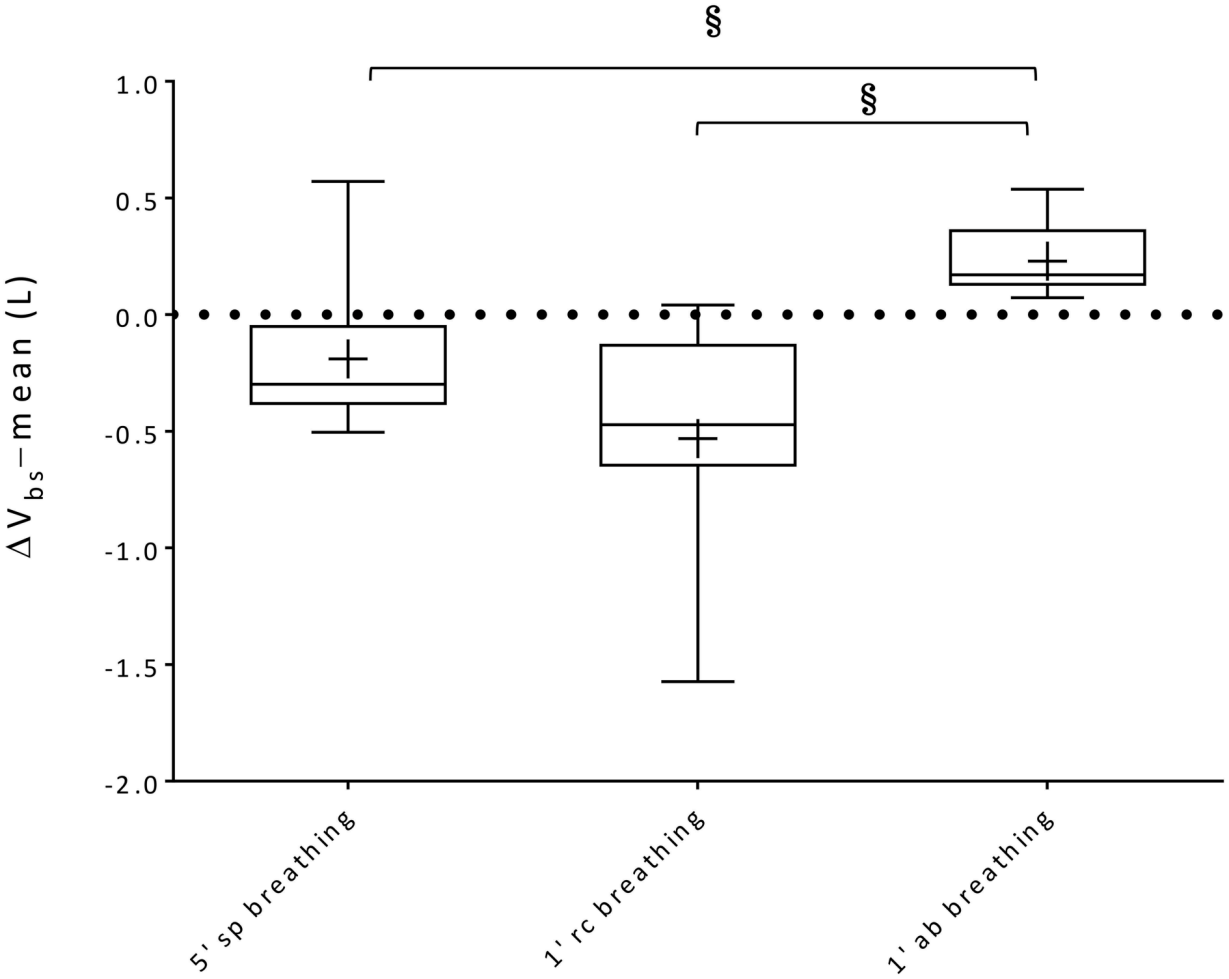
**Box-and-whisker plots illustrating ΔV_bs_-mean during 5′ spontaneous breathing, 1′ rib cage breathing and 1′ abdominal breathing mode exercise respectively**. Central line, box and whisker limits represent median, interquartile range and minimum and maximum values of the data, respectively. + represents the mean value; ^§^*p* < 0.05 vs. abdominal breathing mode.

Within-breath V_bs_ swings and variations of average V_bs_ values (ΔV_bs_-mean) during spontaneous, rib cage and abdominal breathing modes are presented in Figures 6, 7, respectively. Mean V_bs_ swings, were calculated as the mean difference between maximum and minimum values for each series of breaths analyzed, for quiet breathing and exercise respectively. During exercise, each subject showed a consistent increase in within-breath V_bs_ swings compared to pre-exercise (0.26 ± 0.26, 0.28 ± 0.25, 0.33 ± 0.22 L, respectively during spontaneous, ribcage and abdominal modes, *p* < 0.05, *p* < 0.01, and *p* < 0.001). On average, ΔV_bs_-mean decreased by 0.19 ± 0.31 L and 0.53 ± 0.51 L (p = n.s., *p* < 0.01) respectively during spontaneous and rib cage modes, indicating blood shifts from the extremities into the trunk. Conversely during abdominal exercise ΔV_bs_-mean tended to increase in average by 0.23 ± 0.16 L (*p* < 0.01), indicating blood shifting from the trunk to the extremities (Figure 7) and showing complex V_bs_ patterns, resulting from the specific breathing patterns used by the subjects. This led to significant differences in blood redistribution. On average 0.16 ± 0.33 L and 0.48 ± 0.55 L were drawn into the trunk, presumably into the rib cage, in spontaneous and rib cage breathing mode, respectively (p = n.s., spontaneous vs. rib cage mode) while during abdominal mode 0.22 ± 0.20 L of blood (*p* < 0.01, both rib cage and spontaneous vs. abdominal) was pushed out of the trunk.

## Discussion

We investigated how changes in breathing mode, during exercise, by acting on intrathoracic and abdominal pressure variations, influence blood shifting between different body compartments, and in particular in and out of the trunk. We expected that during exercise, compared to rest, splanchnic venous blood would be mobilized, that the amount of blood displaced within each breathing cycle would increase, and that the direction and amount of this blood displacement would depend on the relative contributions of the actions of the diaphragm, the rib cage and abdominal muscles. The main results can be summarized as follows: (a) for the first time the extent of blood volume shifting between the trunk and the extremities was non-invasively quantified during a form of leg exercise; (b) compared to pre-exercise baseline conditions, during exercise, while in spontaneous breathing mode, on average 160 ml of blood were pulled into the trunk from the extremities; (c) during abdominal mode, when the subjects breathed predominantly using the diaphragm and the abdominal muscles, about 225 ml was shifted out of the trunk to the extremities; (d) conversely, during rib cage mode, when the subjects voluntary emphasized the use of inspiratory rib cage muscles, about 478 ml was pulled into the trunk from the extremities; (e) at baseline, intra-breath V_bs_ amounted to 134 ml on average, slightly higher as reported before, probably because of slight hyperventilation, while during exercise it increased to about 420 ml, independently of the breathing mode, increasing inferior vena cava venous return from 2.5 L/min at baseline to 11.4 L/min during exercise. It follows that depending on the partitioning between respiratory muscles for the act of breathing, the distribution of blood between trunk and extremities can vary by up to 1 L and that during exercise the abdominal muscles together with the diaphragm might play a role of an “auxiliary heart.”

### Measuring blood shifting

Other studies examined the influence of the mechanics of breathing on the circulatory system, mainly qualitatively, evaluating the effects of within-breath changes of intrathoracic and abdominal pressures on venous return and blood redistribution from the splanchnic region (Flamm et al., 1990; Miller et al., 2005a,b). Aliverti et al. (2009) were the first to quantify this blood redistribution. They demonstrated that the increase in abdominal pressure during the descent of the diaphragm leads to substantial quantities of blood (V_bs_) shifted from the trunk and presumably the splanchnic venous reservoir, to the extremities. These observations support the notion of a circulatory role for the diaphragm, as also illustrated by the effects of phrenic pacing of the diaphragm on blood shifting reported by Roos et al. (2009).

We now quantified the effects of different breathing modes on blood volume redistribution between the trunk and the extremities during physical activity of moderate intensity. Three breathing patterns were chosen deliberately to vary the contribution of different muscles groups, in order to attain various combinations of breath-by-breath changes in intra-thoracic and abdominal pressures. We chose this approach to complement the results from Aliverti et al. (2010) who measured blood redistribution during expulsive maneuvers while sitting at rest. They found that the circulatory effect of the diaphragm was enhanced when supported by the action of abdominal muscles, and resulted in a consistent “stroke volume” of V_bs_ (on average 350 ml), corresponding to blood coming from the splanchnic circulation (presumably mostly from the liver), through the hepatic vein into the inferior vena cava downstream toward the heart.

In the present study, the three breathing modes, by acting differently on breath-by-breath changes in intra-thoracic and abdominal pressures, affected V_bs_ in two ways. According to Aliverti et al. (2009, 2010), during abdominal mode breathing and expulsive maneuvers, V_bs_ represents blood coming from the splanchnic vascular bed returning to the right heart to be then injected into the systemic circulation, since the venous return between the femoral vein and the inferior vena cava at the inlet of hepatic vein is temporarily stopped by the disappearance of the pressure gradient between those points (Miller et al., 2005b; Uva et al., 2010). Conversely, during rib cage mode breathing, a decrease in esophageal pressure decreases right atrial pressure, thus increasing the pressure gradient between the femoral veins and the right heart, facilitating venous return from the lower extremities. In this case what we measured as ΔV_bs_-mean is mostly venous return from the extremities. In this perspective our results complete and extend previous reports by others (Miller et al., 2005b; Aliverti et al., 2010).

### Respiratory modulation of splanchnic blood shifting

In our study, most subjects, when switching from pre-exercise baseline to exercise while breathing spontaneously, predominantly recruited their inspiratory ribcage muscles, which resulted in an increase in end-inspiratory rib cage volume, while end-expiratory abdominal volume did not differ significantly from that at pre-exercise baseline (see Figure 5). The breathing patterns used by the subjects during this breathing mode, led to substantial variation in V_bs_ (see Figure 6) but the prevailing effect with regard to V_bs_-mean was a drop. This result indicates that blood was being pulled into the trunk, suggesting increased venous return toward the right heart. During rib cage mode exercise, when the subjects emphasized the use of inspiratory rib cage muscles, while relaxing the diaphragm and abdominal muscles, this effect was amplified (ΔV_bs_-mean amounted to 0.19 ± 0.31 L (p = n.s.) and 0.53 ± 0.51 L (*p* < 0.01), for spontaneous and rib cage exercise, respectively). The stronger contraction of the inspiratory rib cage muscles, by lowering intra-thoracic pressure during inspiration likely resulted in a more pronounced right atrial to inferior vena cava pressure difference, thus facilitating venous return, confirming earlier observations (Guyton et al., 1955; Miller et al., 2005a,b). This more pronounced effect during rib cage mode breathing in our set-up could be explained by a moderate degree of chest wall hyperinflation, likely due to a persistent contraction of the inspiratory muscles during the expiratory phase of the breathing cycle (see Figure 5), that would have led to a decreased P_pl_ for a more prolonged time. These findings during rib cage mode and also somewhat during spontaneous mode are qualitatively similar to what Flamm et al. (1990) found in their study, demonstrating a consistent blood volume shift from the splanchnic reservoir and the lower extremities to the pulmonary circulation during zero load spontaneous upright exercise.

By contrast, during abdominal mode breathing the situation was reversed. When the subjects activated their abdominal expiratory muscles, tidal volume increased due to a decrease of end-expiratory abdominal volume, while rib cage end-inspiratory volume remained nearly constant (see Figure 5). The contraction of the diaphragm and of the abdominal muscles, respectively during inspiration and expiration, presumably increased baseline abdominal pressures and pressure swings, while average pleural pressure remained rather invariant. This led to a significant increase both in V_bs_ swings and in the amount of blood displaced out of the trunk, since the femoral venous return toward the trunk was likely stopped by the increase in abdominal pressure (Willeput et al., 1984; Miller et al., 2005a). The source of the V_bs_ must then have been the splanchnic venous reservoir (Aliverti et al., 2009).

We acquired esophageal and abdominal pressures in three of our subjects but do not present the results in detail because of the small number of observations and the variability between subjects. But although abdominal and pleural pressure patterns differed between subjects during the various breathing modes, it appeared that the V_bs_ bimodal shape uniquely tracked the individual changes in abdominal and pleural pressures. In particular, when breathing in abdominal mode during stepping exercise, in all three an increase in abdominal pressure swings was found while pleural pressure swings remained similar to those during pre-exercise baseline breathing, which led to a series of repeated expulsive-like maneuvers. This cyclical increase in abdominal pressure (~20 cmH_2_O on average) led to a tidal blood volume of 460 ml, presumably through the hepatic vein, and a global splanchnic output of about ~12 L/min.

Conversely, when the subjects started to exercise while breathing in rib cage mode, a marked increase in pleural swings was evident, while the abdominal pressure swings were determined by the passive movement of the diaphragm, which transmitted pleural pressure variations to the abdominal content. In this case, as demonstrated by Miller et al. (2005b), augmenting the inspiratory negative intrathoracic pressure excursions resulted in a more negative right atrial pressure. The greater inspiratory facilitation of venous return resulted in an average 470 ml of blood pulled into the rib cage for each breath, which led a total flow drawn from the extremities of about ~11 L/min.

The intra-breath increase in V_bs_ swings during exercise was found during all three breathing modes, suggesting a dynamic impact on venous return through the inferior vena cava to the heart. A “stroke volume” of about 420 ml during all breathing modes was found in agreement of what has been previously published by Aliverti et al. during intermittent expulsive maneuvers (Aliverti et al., 2010). A further bimodal time course of V_bs_ was found during all breathing modes. During abdominal exercise it seemed tracked only by the changes in abdominal pressure, suggesting that the V_bs_ pattern was driven mostly by the diaphragm, which is the only muscle acting on the abdominal compartment during inspiration, and by the abdominal muscle contraction during expiration. By contrast, during rib cage breathing mode, the biphasic V_bs_ pattern was determined by the large pleural pressure swings, while abdominal pressure variations were kept similar to those at pre-exercise baseline.

These results support previous findings, which suggest that the respiratory muscles, acting on abdominal and pleural pressure dynamics, have an important cardiovascular function. Based on our oxygen uptake measurements we can estimate (Beck et al., 2006) that cardiac output while sitting in the body box at baseline was 6.4 ± 0.6 L/min and during exercise in spontaneous breathing mode increased to 10.2 ± 1.4 L/min, suggesting that the above mentioned 11 L/min somewhat overestimates inferior venous cava venous return, since superior venous cava return obviously was not nil.

### Rib cage, abdominal, and lower limb blood volume redistribution

Our results contribute quantitative evidence for important respiratory modulation of V_bs_ partitioning during exercise. As previously described, we believe that what we report as V_bs_ is the volume of blood moved between the splanchnic vascular bed, rib cage, lungs and extremities. Since with our set-up we measured the variation in the total chest wall volume, we cannot precisely estimate the blood that during exercise is shifting between the splanchnic bed, and thus the abdomen, and the pulmonary circulation and the rib cage, because they belong to the same compartment.

Upon starting the stepping activity, while breathing in abdominal mode, we measured a positive blood shift from baseline, indicating that blood was shifted out of the trunk to the extremities. Aliverti et al. (2010) measured cardiac output and blood pressure during expulsive maneuvers and demonstrated that V_bs_ is of splanchnic venous compartment origin. In the present study we therefore believe that the V_bs_ we measured must be the result of splanchnic vascular outflow toward the heart minus concomitant arterial outflow toward the extremities. Within each breath the splanchnic blood reservoir would not be completely refilled, resulting into a significant redistribution of blood between splanchnic bed, exercising muscles (i.e., leg muscles and respiratory muscles) and lungs (Harms et al., 1997).

This hypothesis is consistent with previous studies which proposed the splanchnic vascular bed as a significant venous reservoir for blood redistribution during exercise (Rowell, 1974). During rib cage mode breathing, we found on average 478 ml of blood coming into the trunk from the extremities. Contraction of the rib cage inspiratory muscles, by making average pleural pressure more negative, thus increased venous return to the right heart. Our data do not permit partitioning of where exactly this extra venous return then went. Presumably part was used for lung capillary recruitment (Johnson et al., 1960; Flamm et al., 1990) while part of it left the trunk through the big arteries into the extremities. This effect, which is expected to be more important during rib cage breathing mode, might contribute in part to the increase in rib cage blood volume compared with the other breathing modes. Part of the blood may have gone to the respiratory muscles, which require an increase in blood flow because of increased activity. In our study rib cage mode exercise led to an increase in ventilation and a decrease in P_ET_CO_2_ indicating increased breathing workload compared to abdominal breathing mode. This would be consistent with data obtained in an animal model showing an increase in perfusion of the respiratory muscles (diaphragm and intercostal muscles) and a decrease in intestinal blood flow (Fixler et al., 1976). In several animal studies this redistribution of cardiac output seems to reflect the increase in peripheral vascular resistance related to the concomitant decrease in respiratory vascular resistance (Fixler et al., 1976; Robertson et al., 1977; Sheel et al., 2001).

### The role of skeletal and respiratory muscle pumps

It is thought that lower limb muscle contraction contributes to venous return (Laughlin, 1987; Hamann et al., 2003; Tschakovsky and Sheriff, 2004) facilitating the propulsion of the blood from the skeletal muscle vasculature. In the present study, we have no data on changes in blood flow by the action of the skeletal muscle pump and its relative role with respect to the respiratory muscle pump. During mild calf contraction exercise, Miller et al. (2005b) found a biphasic within-breath modulation of femoral venous blood flow, which seemed to be in phase with the rhythmic muscular contraction (Figure 4 of their paper). Likewise the biphasic within-breath V_bs_ shape we observed might be attributed to the cyclical contraction of the skeletal muscle pump during the exercise. But since the legs were exercised in the same manner in all breathing modes exercise and the biphasic V_bs_ pattern seemed to be determined by the individual variation of abdominal and esophageal pressure variations, we assume that the large variations in blood flow partitioning that we found between abdominal and rib cage modes should be mainly attributed to the action of the different respiratory muscles involved.

Our study thus lends support to what previously was found by Miller et al. who demonstrated that the respiratory muscle pump has a prevailing effect during exercise rather than the skeletal muscle pump (Miller et al., 2005b). The respiratory muscle pump, enhancing negative intrathoracic pressure, draws blood toward the heart and increases venous return, stroke volume and cardiac output, corroborating its importance in enhancing systemic circulation and vital organ perfusion, also during critical conditions such as hemorrhage (Hodges et al., 2001; Convertino et al., 2004; Yannopoulos et al., 2006; Poh et al., 2014).

### Potential implications of blood redistribution during whole body exercise

Our findings open interesting perspectives on the role of the “abdominal pump” during various types of physical activity, for example during walking or running, when the diaphragm and the abdominal muscles contribute to postural control of the trunk through elevation of the intra-abdominal pressure and the skeletal muscle pump forces blood centrally. In this study we have expanded the findings reported by Aliverti et al. (2010) during expulsive maneuvers and Miller et al. (2005b) during light exercise, demonstrating that during submaximal upright exercise, the respiratory muscles acting on pleural and abdominal pressure swings, are able to partition blood flow between the rib cage and pulmonary circulation, the working muscles, and the abdominal venous compartment.

These results have important implications in whole-body exercise, as for example walking or running when also the upper limb will be involved in the exercise and the abdominal pressure would increase in order to increase the lumbar spine stability (Cholewicki et al., 1999a,b; Hodges and Gandevia, 2000). Ainsworth and colleagues demonstrated in exercising horses a locomotory-associated modulation of the abdominal and intrathoracic pressure (Ainsworth et al., 1996). It remains to be investigated if also during human running such phase-coupling relationships between limb movement and breathing will influence the circulatory function of respiratory muscles and thus the subsequent blood volume partition between different compartments.

## Limitations

When interpreting the results of our study one should keep in mind some limitations. First, because of the complexity of our experimental setup we decided to refrain from measuring cardiac output (heart rate and stroke volume), which precludes any strong conclusions on the effects of the observed blood shifts on cardiac function. Second, what was measured in this study as V_bs_ was considered to represent liquid (mainly blood) moved between the trunk and the extremities. Since we measured the total V_cw_ using OEP, we could not determine the amount of blood shifted between the rib cage and the abdominal compartments, limiting our assertions on splanchnic blood volume recruitment. Third, in this study we have not investigated the effect of the specific breathing modes at rest in comparison to during exercise in the same subject. Fourth we do not report gastric and esophageal pressures since these were only acquired in 3 subjects and equivocal. Fifth, because of the complexity of thermodynamic control inside the body box exercise duration had to be kept short. Even though our conditioning system was able to keep temperature quite stable during exercise, it did not allow complete control of humidity. We had to develop a mathematical method to correct for this drift. In order to avoid all the possible problems related to the influence of filtering on the signal baseline, we therefore analyzed the blood shifts only in terms of variations of baseline and tidal volumes respectively. Sixth, the respiratory maneuvers were difficult to perform. Several of the subjects had difficulties in performing the complex respiratory maneuvers by changing their breathing modes correctly during exercise. The various attempts were classified according to the variations in compartmental volumes, which led to various numbers of repetitions between breathing modes for some subjects. Finally, because of the complexity of the maneuvers there were missing values precluding the use of simple ANOVA. We therefore chose generalized linear mixed modeling, but in the worst case the proportion of missing values was one third, limiting the robustness of the statistical analysis.

## Conclusions

In summary, for the first time the volume of blood redistributed between the trunk and the extremities was quantified non-invasively and continuously during exercise. The amount and the partial redistribution of this blood depended on the activation of rib cage, diaphragm and abdominal muscles on a breath-by-breath basis. In particular we found that during abdominal exercise the action of the diaphragm and abdominal muscles, increasing the abdominal pressure can shift blood from the extremities, while the rib cage inspiratory muscles' action facilitates blood shifting toward the pulmonary circulation. Depending on the partitioning between respiratory muscles for the act of breathing, the distribution of blood between trunk and extremities can vary by up to 1 L. We conclude that during exercise the abdominal muscles assist the diaphragm in its role of an “auxiliary heart.”

### Conflict of interest statement

The authors declare that the research was conducted in the absence of any commercial or financial relationships that could be construed as a potential conflict of interest.

## Abbreviations

DBP: double body plethysmography
DWT: discrete wavelet transformation
OEP: opto-electronic plethysmography
P_ab_: abdominal pressure
P_pl_: pleural pressure
P_ET_CO_2_: end-tidal CO_2_ pressure
V_b_: WBP-measured body volume
V_bs_: volume of blood shifting between the trunk and the extremities
V′CO_2_: carbon dioxide expired
V_ab_: OEP-measured abdominal volume
V_abEE_: end-expiratory abdominal volume
V_abEI_: end-inspiratory abdominal volume
V_cw_: OEP-measured chest wall volume
V_cwEE_: end-expiratory chest wall volume
V_cwEI_: end-inspiratory chest wall volume
V_E_: minute ventilation
V′O_2_: oxygen uptake
V_rc_: OEP-measured rib cage volume
V_rcEE_: end-expiratory rib cage volume
V_rcEI_: end-inspiratory rib cage volume
V_T_: chest wall tidal volume
V_tr_: OEP-measured trunk volume
WBP: whole body plethysmography.
